# Supplementary material for: Protocol for the OCAY study: a cohort study of orphanhood and caregiver loss in the COVID-19 era to explore the impact on children and adolescents
Source: BMJ Open. 2023 Jun 1;13(6):e071023. doi: 10.1136/bmjopen-2022-071023 (PMC10254791; doi:10.1136/bmjopen-2022-071023)
Supplement: Author's manuscript [file bmjopen-2022-071023.draft_revisions.pdf]

BMJ Open

BMJ Open is committed to open peer review. As part of this commitment we make the peer review history of every article we publish publicly available.

When an article is published we post the peer reviewers' comments and the authors' responses online. We also post the versions of the paper that were used during peer review. These are the versions that the peer review comments apply to.

The versions of the paper that follow are the versions that were submitted during the peer review process. They are not the versions of record or the final published versions. They should not be cited or distributed as the published version of this manuscript.

BMJ Open is an open access journal and the full, final, typeset and author-corrected version of record of the manuscript is available on our site with no access controls, subscription charges or pay-per-view fees (http://bmjopen.bmj.com).

If you have any questions on BMJ Open's open peer review process please email info.bmjopen@bmj.com

BMJ Open

Protocol for the OCAY study: A cohort study of orphanhood and caregiver loss in the Covid-19 era to explore the impact on children and adolescents

Journal:	*BMJ Open*
Manuscript ID	bmjopen-2022-071023
Article Type:	Protocol
Date Submitted by the Author:	19-Dec-2022
Complete List of Authors:	Steventon Roberts, Kathryn; University of Oxford, Department of Social Policy and Intervention ; University College London, Institute for Global Health<br>Du Toit, Stefani; Stellenbosch University<br>Mawoyo, Tatenda; Stellenbosch University<br>Tomlinson, Mark; University of Stellenbosch, Institute for Life Course Health Research<br>Cluver, Lucie; University of Oxford, Social Policy and Intervention<br>Skeen, Sarah; Stellenbosch University<br>Sherr, Lorraine; University College London
Keywords:	COVID-19, Public health < INFECTIOUS DISEASES, Community child health < PAEDIATRICS, PSYCHIATRY, PUBLIC HEALTH

SCHOLARONE™
Manuscripts

Protocol for the OCAY study: A cohort study of orphanhood and caregiver loss in the Covid-19 era to explore the impact on children and adolescents

Kathryn J. Steventon Roberts[1,2]*

Stefani Du Toit[3]*

Tatenda Mawoyo[3]*

Mark Tomlinson[3,4]

Lucie Cluver[1,5]

Sarah Skeen[3]

Lorraine Sherr[2]

*Joint contributions

Corresponding author: Lorraine Sherr l.sherr@ucl.ac.uk

[1]Department for Social Policy and Intervention, University of Oxford, Oxford, UK

[2]Institute for Global Health, University College London, London, UK

[3]Institute for Life Course Health Research, Department of Global Health, Stellenbosch University, South Africa

[4]School of Nursing and Midwifery, Queens University, Belfast, UK

[5]Department of Psychiatry and Mental Health, University of Cape Town, Cape Town, South Africa

For peer review only

Abstract

Introduction: Globally, no person has been untouched by the COVID-19 pandemic. Yet, little attention has been given to children and adolescents in policy, provision, and services. Moreover, there is a dearth of knowledge regarding the impact of COVID-19 associated orphanhood and caregiver loss on children. This study aims to provide early insights into the experience children and adolescence experiencing orphanhood or caregiver loss in South Africa.

Methods and Analysis: Data will be drawn from a quantitative longitudinal study undertaken initially in Cape Town, South Africa. A sample of children between the ages of 9 -16 years, experiencing parental or primary caregiver loss from COVID-19 will be recruited together with a comparison group of children in similar environments who did not experience loss. The study aims to recruit at least 500 children in both groups. Mental health and well-being among children will be explored through the use of validated and study specific measures. Participants will be interviewed at two time points, with follow-up data being collected 12 – 18 months after baseline. A combination of analytical techniques (including descriptive statistics, regression modelling and structural equation modelling) will be utilised to understand the experience and inform future policy and service provision.

Ethics and dissemination: This study received ethical approval from the Health Research Ethics Committee at Stellenbosch University (N 22/04/040). Results will be disseminated via academic and policy publications, as well as national and international presentations including high-level meetings with technical experts. Findings will also be disseminated at a community level via various platforms.

Strengths & Limitations

- Globally, this study will be the first empirical investigation of the impacts of COVID- 19 associated caregiver loss on the wellbeing of children and youth.
- A key strength of the study is the broad array of domains covered by the questionnaire used within this study and, the use of validated and standardised scales.
- The proposed research will generate evidence to inform further research, effective policy as well as the development of innovative and appropriate interventions for at-risk orphans and vulnerable children.
- Future research should explore this key issue in other countries with high burden of COVID-19 mortality.

For peer review only

Background

The COVID-19 pandemic has had a dramatic impact on people across the world. The mortality rate from the virus has been high, with no country untouched, and notable large-scale deaths among parents and caregivers of children and adolescents – an estimated 10.5 million children and adolescents have experienced the death of a parent or caregiver [1, 2]. Yet, there has been little attention focused on the children and adolescents experiencing the death of parents and caregivers and the resulting consequences for their health and wellbeing. Orphanhood is defined as the death of one or both parents [3]. Orphanhood has previously been associated with an increased risk of poor mental health, violence and abuse exposure, unstable home environments, school disruption, early sexual debut, and poverty [4-11]. Extending beyond parents, alternative caregivers, such as grandparents are often key in providing essential care for children and adolescents. Due to the multigenerational nature of households, it is also important to consider caregiver loss as grandparents are often primary caregivers and were the most vulnerable to COVID-19 [1, 2, 12-14].

There needs to be a clear understanding and tracking of the nature of orphanhood and caregiver loss so that provision for children can be accurately tailored [15]. The occurrence of COVID-19 orphanhood may well be layered onto pre-existing social systems where children already experience a multitude of social challenges and other forms of loss e.g., due to HIV or Tuberculosis [16]. However, there may be elements of orphanhood and caregiver loss associated with COVID-19 that are new and yet undescribed and uncharted. Similarities from previous large-scale parental loss experiences such as HIV/AIDS, Ebola, war, and disaster needs to be understood while this new group of children and youth need to be linked to existing provision.

Undertaken within South Africa, the OCAY study is to the best of our knowledge, the first empirical study to explore the impact of orphanhood and caregiver loss on the wellbeing of children and adolescents. Children and adolescents who have experienced the loss of a caregiver due to COVID-19 and a comparison group of children and adolescents who have not experienced loss will be recruited to the study. Objectives of the study are four-fold; 1) to determine if mental health outcomes (positive/negative) differ between children and adolescents who have experienced a COVID-19 associated caregiver death, and children and adolescents who have not experienced a COVID-19 associated caregiver death; 2) to explore the impact of COVID-19 associated caregiver death on health and wellbeing outcomes of children, and service use (comparing those with and without a caregiver loss); 3) to explore how mental health outcomes (positive/negative) among children and adolescents who have experienced orphanhood and caregiver loss change over time (utilising follow up data); 4) to examine associations between access to services (such as COVID-related grants, bereavement referrals) and outcomes for children and adolescents.

Methods and study design

Study setting

The study will predominantly take place in Khayelitsha, which is an impoverished peri-urban settlement on the outskirts of Cape Town, South Africa. Khayelitsha is the third largest township in South Africa, with an estimated population of between 400 000 and 750 000 [17]. The majority of residents live in overcrowded informal housing with shared communal water taps and inadequate sanitation arrangements [17]. According to the 2011 Census, the average annual household income was R20 000 (approximately $1,200) [18]. The high unemployment rate, approximately 50%, underpins the high rate of poverty [19]. Khayelitsha experience significant high levels of crime and

For peer review only

lack consistent and effective police services [20]. As existing studies forms part of our recruitment approach, participants residing in other parts of South Africa will also be considered for inclusion.

Participant recruitment

The study seeks to identify at least 500 children and adolescents aged between 9 – 16 years of age, that have been orphaned or lost a primary caregiver due to COVID-19 (defined as COVID-19 infection or likely COVID-19 infection). A similar proportion (n=500) of children and adolescents (9-16 years of age) who have not lost as primary caregiver due to COVID-19 will be recruited as a comparison group. the non-affected group. To identify the sample a mosaic of five strategies will be utilised: (1) *Existing studies* – participants included in previous studies conducted by the groups Primary Investigators (PI) and Co-primary investigator's (Co-PI) will be contacted to assess eligibility [21-25]; (2) *Community links* –neighbourhoods within our study setting often have street committees, and these committees will be utilised to identify eligible participants. Alternatively, identification could take place by door-to-door recruitment; (3) *Schools* – following permission from the Western Cape Education Department (WCED), primary and high schools within the area will be approached for recruitment; (4) *Non-governmental organisations (NGOs)* – NGOs within our study setting, rendering services to children and adolescents, will be utilised; (5) *Snowball sampling* – participants recruited into the study will be provided with the opportunity to refer other potential participants.

Patient and Public Involvement

It was not appropriate or possible to involve patients or the public in the design, or conduct, or reporting, or dissemination plans of our research at the time of writing this protocol.

Study measures

All participants will be interviewed at two time points – baseline and 12-18 months later. A study specific inventory, adjusted for age-appropriate completion will be constructed. The components will use validated measures which have been used in five previous studies undertaken with children and adolescents within South Africa (the Young Carers study, the Mzansti Wakho study, the HEY BABY study, the Child Community Care Study, and the Adolescent Bereavement Initiative). Given the broad extent of the impact of orphanhood on the lives of the children and adolescents, measures will explore multiple aspects of life including: (1) Demographic and social circumstance factors; (2) Mental health (including depression, anxiety, posttraumatic stress and suicidality) and wellbeing; (3) COVID-19 experience; (4) Bereavement experience; (5) Care and subsequent care experience; (6) Experience of support & provision (7) Health experience; (8) Education and school experience; (9) Behavioural risk; (10) Violence exposure, stigma and bullying experience; and (11) Parenting and caregiving experience. Measures will be piloted and refined/adapted based on participant feedback. See Table 1 for an overview of measures to be utilised within the study.

For peer review only

Table 1. Study measures for inclusion within the OCAY study

Domain	Measures	Example items & response categories	Examples of previous use and/or validation within LMIC
**Demographic & social circumstance factors**			
Demographic information	Child age, biological sex, gender, and basic demographic information will be obtained from items utilizing the South African Census [26].	-	-
Anthropometric measurements	Height and weight will be obtained to assess physical development	-	-
Household food security	Study specific items adapted from the household food insecurity access scale (10 items) which measures food security in the previous 30 days	E.g., Did you worry that your household would not have enough food?<br><br>Response: Never/Rarely/Sometimes/Often	Marlow et al (South Africa)[27]<br><br>Knueppel et al (Tanzania) [28]<br><br>Kabalo et al (Ethiopia) [29]
Socioeconomic status	Do you have running water? Do you have electricity? Do you have a flush toilet?	E.g., Do you have running water at your house?<br><br>Response: Yes/No	Du Toit et al (South Africa)[30]
	Socioeconomic status will also be measured by measuring access to the top 8 socially-perceived necessities for children, as identified by the Centre for South African Social Policy in the 'Indicators of poverty and social exclusion project' [31] and endorsed by over 80% of the South African population in a nationally-representative survey (the South African Social Attitudes Survey 2006) [32]. These include items such as 'enough clothes to keep you warm and dry'.	Please tick all of the things that you can afford at home….<br><br>Response: E.g., 3 meals a day, school fees	Cluver et al (South Africa)[33]<br><br>Steventon Roberts et al (South Africa)[34]
	Cash grant receipt	E.g., Are you or anyone in your household receiving any grants?	-
	Household employment	E.g., How many people in your household are employed?	-
Household information	Study specific questions on household arrangements & living circumstances	E.g., What type of house do you live in?<br><br>Response: E.g., House made of bricks on a separate yard/block of flats	-
**Health**			
COVID-19	Study specific questions on experience of COVID-19 including caregiver death, COVID-19 infection within the household, experience of lockdown and presence of long COVID	E.g., Were you or anyone in your household infected with COVID-19?<br><br>Response: Yes/No	-
HIV	Study specific questions on household HIV Status, testing, status, and treatment	E.g., Is anyone in your household living with HIV?<br><br>Response: Yes/No/Don't know/Decline to answer	-
Overall health status	An adapted subscale from the International Classification of Functioning Disability and Health (3-items) will be utilized to assess self-reported health within the previous 12 months[35]	E.g., In the last 12 months, did you have difficulty remembering things or following a story or conversation?<br><br>Response: No, no difficulty at all/Yes, some difficulty/Yes, a lot of difficulty/Cannot do it at all	-
Sexual and Reproductive Health	Study specific questions adapted from the National Survey and risk behaviour amongst South Africans [36] will be used to explores sexual and reproductive health. Items focus on sexual activity, age of sexual debut, contraception use, transactional sex, substance use during sexual activity, pregnancy & sexual abuse	E.g., Are you currently in a sexual and/or romantic relationship or have you ever been in a sexual and/or romantic relationship?<br><br>Response: Yes/No	-
Health service utilisation	Study specific questions regarding what health services have been accessed by the participant in the previous 12 months	E.g., Which of the following health services have you accessed in the last 12 months?	-
**Mental Health**			
Depressive symptoms	The Child Depression Inventory Short Form (10 items; CDI-SF) [37] was used to assess depressive symptomology within the previous two weeks.	E.g., Select the statement which best describes how you have felt in the past 2 weeks.<br><br>Response: Nothing will ever work out for me/ I am not sure if things will work out for me/ Things will work out for me OK	Suliman et al (South Africa) [38]<br><br>Cluver et al (South Africa) [33]<br><br>Sherr et al (South Africa, Malawi, Zambia) [39]<br><br>Roberts et al (South Africa) [40]
	The Patient Health Questionnaire – 9 (9 items; PHQ-9) [41] was used to assess depressive symptomology within the previous two weeks.	E.g., Have you felt down, depressed, irritable, or hopeless?<br><br>Response: Not at all/Several days/More than half the days/Nearly every day	Aggarwal et al (South Africa) [42]<br><br>Rakshasa-Loots et al (South Africa) [43]<br><br>Chibanda et al (Zimbabwe) [44]
Anxiety symptoms	The Generalized Anxiety Disorder-7 (7 items; GAD-7) [45] was used to assess anxiety symptoms in the previous two weeks	E.g., Have you felt nervous, anxious or on edge?<br><br>Response: Not at all/Several days/More than half the days/Nearly every day	Kigozi et al (South Africa) [46]<br><br>Adjorlolo et al (Ghana) [47]<br><br>Roberts et al (South Africa) [40]<br><br>Chibanda et al (Zimbabwe) [44]<br><br>Mughal et al (LMIC) [48]
Posttraumatic stress symptoms	A single item informed by the Child PTSD checklist was used to explore posttraumatic stress symptoms among those children and adolescents' who had lost a caregiver	How stressed have you been since the death of your caregiver?<br><br>Response: Very/A little/No more than usual	Boyes et al (South Africa) [49]<br><br>Roberts et al (South Africa) [40]
Suicidality and self-harm	Mini International Psychiatric Interview for Children and Adolescents Suicidality (5 items; MINI-Kid) [50] was used to assess suicidality and self-harm behaviours in the previous month	E.g., In the past month did you wish you were dead?<br><br>Response: Yes/No	Sheehan et al & Lecrubier et al (Global) [51, 52].

For peer review only

			Cluver et al (South Africa) [33]<br><br>Roberts et al (South Africa) [40]
Self-esteem	The Rosenberg Self-Esteem Scale (10 items) [39] was used as an assessment of child and adolesent self-esteem	E.g., I feel that I have a number of good qualities<br><br>Response: Strongly agree/Agree/Disagree/Strongly Disagree	Sherr et al (South Africa, Malawi, Zambia) [39]
Access to mental health services	A single study specific item to assess whether children and adolescents have access to mental health services	Do you have access to mental health services?<br><br>Response: Yes, needed and accessed/Yes, but didn't need so didn't access/No, needed but couldn't access /No, didn't need and didn't access	-
**Bereavement experience**			
Grief	The Core Bereavement Items – Grief sub-scale (5 items) will be used to assess grief intensity [53]	E.g., Do reminders of X such as photos, situations, music, places etc. cause you to feel longing for X?<br><br>Response: A lot of the time/Quite a bit of the time/A little bit of the time/Never	Thurman et al (South Africa) [54]
**Parenting & caregiving experience**			
Parenting experience	The Alabama Parenting Questionnaire (16 items) will be used to identify experience of positive parenting, caregiver supervision, experience of caregiver discipline within the previous two months[55]	E.g., Thinking about the person who looks after you the most, your parent or caregiver says that you have done something well…<br><br>Response: Never/Rarely/Sometimes/Often/Always	Cluver et al (South Africa)[56]<br><br>Elgar et al (Global) [55]
Experience of caregiving and responsibility for household tasks	The Young Carers Tasks & Outcomes Questionnaire (23 items [57] was adapted to assess children and adolescents experiences of responsibility for household tasks	E.g., In the past week has you washed other people's clothes?<br><br>Response: Yes/No<br><br>How many days a week do you do this?	Lane et al (South Africa) [58]
**Support interventions & provision**			
Organisational support	Study specific questions on support from organisations e.g., support from social services, healthcare workers	E.g., Do you have access to an organisation that provides you with any support services?<br><br>Response: Yes/No	-
Community provision	Study specific questions on community provisions	E.g., Are you a member of any youth and/or health organisations, political or activist groups?	-
**Education & School experiences**			
Education	Study specific questions on school enrolment, being in the correct class for age, educational attainment, education aspirations and school dropout.	E.g., Are you currently enrolled in school?<br><br>Response: Yes/No	-
**Behavioural risk**			
Behavioural problems	The Child Behaviour Checklist: Rule-breaking subscale (17 items) was used to assesses behavioural problems in the previous 6 months[59]	E.g., I drink alcohol to have fun, without my caregivers knowing or approving<br><br>Response: Not true/Somewhat or sometimes true/Very true or often true	Sherr et al (South Africa, Malawi, Zambia) [39]
**Violence exposure, stigma & bullying**			
Violence exposure	Study specific questions adapted from the UNICEF measures for national level monitoring of orphans and vulnerable children [60] and the Parent-Child Conflict Tactics Scales were used to assess household, domestic, and school violence	E.g., How often has anyone in your family or living in your home or someone at school used a stick, belt, or other hard item to hit you?<br><br>Response: Weekly/Monthly/At least once a year/Has happened but not in the last year/Never	-
Community violence	The child exposure to community violence checklist [61] will be used to identify children and adolescents' exposure to community violence	E.g., Have you ever been hot or attacked outside your home?<br><br>Response: Yes, more than a year ago/Yes, in the last year/Never	Sherr et al (South Africa, Malawi, Zambia) [39]<br><br>Steventon Roberts et al (South Africa) [62]
Stigma	Study specific questions focusing on COVID-19 related stigma	E.g., I have been treated badly because I lost a caregiver to COVID-19<br><br>Response: Not at all/Sometimes/All of the time	-
Bullying	The Social and Health Assessment Peer Victimisation Scale (12 items) was used to examine experiences of bullying in the last 6 months[63]	E.g., In the past 6 months other kids have called me names or swore at me…<br><br>Response: Not at all/Once/2-3times/4 or more times	Boyes et al (South Africa) [64]<br><br>Sherr et al (South Africa, Malawi, Zambia) [39]

Note. Low- and middle-income countries (LMIC)

Power calculation

Power calculations were based on the core mental health measures within the study (depressive/anxiety symptoms). Multiple power calculations have informed this estimate. Previous studies identifying approximately 10% of children and adolescents experience poor mental health [65]. Stratification modelling data based on the methodologies used in Hillis et al. 2022,[2] identified that caregiver loss within South Africa due to COVID-19 has impacted approximately 211 000 children (estimate obtained November 2022; COVID-19 Orphanhood Calculator). Based on this data, a confidence level of 95% and a 2% margin of error the sample size required to detect meaningful difference in the proportion of poor mental health symptomology reported between the sample and general population estimates (Chi-square test) is 861. A second power calculation was undertaken to account for group differences (COVID-19 caregiver loss vs. no COVID-19 caregiver loss) in the core mental health measures (prevalence). Based on identifying a prevalence of 5% poor mental health symptoms (depression/anxiety) among the control group (no COVID-19 associated orphanhood) and 10% prevalence within the population of interest (COVID-19 associated orphanhood), an alpha of 0.05, and 90% power the number of participants required to detect a meaningful difference between groups is 671 (COVID-19 associated orphanhood) vs. 503 (no COVID-19 associated orphanhood). Based on these estimates, and to account for attrition in planned follow-up rounds of data collection, a planned sample of 1,000 children including the number of children and adolescents experiencing COVID-19 associated orphanhood or caregiver loss n=500, and the comparison group of children and adolescents experiencing no COVID-19 associated orphanhood n= 500.

Data collection procedures

Data will be collected by experienced, trained, and supervised data collectors. All data collectors that will be used are highly experienced but will undergo a two-week refresher training workshop in general principles of research ethics, referral procedures, management of difficult situations, obtaining informed consent, and how to administer the questionnaire. Data collectors will also be fluent in English and isiXhosa (the vernacular language of Khayelitsha residents), with prior experience working with vulnerable youth and families in these settings. Assessments will take place at a research centre in Khayelitsha. In the instances where participants are unable to travel to the research centre, assessments will be conducted in a private space at the participants home, school, or NGO. In the instances where participants reside in other parts of South Africa, we will conduct telephonic assessments. Participants will be provided with transportation to and from the venue where assessments are taking place. Prior to starting the interview, caregivers will consent for the participation of their child, and the child will provide informed assent. All measures will be translated into isiXhosa and then back translated into English to ensure the use of accurate and quality translations. Interviews are expected to last between 60-90 minutes. During assessment, participants will be provided with breaks during which they will be served refreshments. All participants are provided with an incentive (shopping voucher) as a means to compensate for their time. Incentives will be provided at each time point. As a means of quality control, the assessments will be audio-recorded, with the permission of the participant. These audio-recordings are reviewed by supervisory staff on a regular basis to ensure consistency in administration between data collectors. Upon completion of the assessments, participants will be provided with a further opportunity to ask any questions or express any concerns.

For peer review only

Ethical considerations:

This study has received ethical approval from the Health Research Ethics Committee at Stellenbosch University (N 22/04/040). This research is designed with child protection principles in mind, and the impact of the questionnaire is a foremost concern. The study will follow ethical procedures used by the researchers in other similar studies working with similar at-risk groups. The questionnaire will be designed to be child friendly. All consent procedures will be conducted prior the commencement of the assessment. Given the young age of some participants and the sensitive nature of the project, all screening and consent processes will be conducted in the homes of the prospective participants. Hopefully, conducting screening and consent procedures in the comfort of the participants' homes will build trust and enhance rapport with both the participants and the caregivers. Caregivers will be welcome to accompany their child to the interview, however, if this is not possible, consent will be obtained prior to the scheduled appointment and permission will be sought to transport the child to and from the research centre or interview venue. Data collectors will explain the interview and assessment procedures to each child, and they will sign an assent form. Caregivers will sign a consent form to provide permission for their child to participate in the study. All participants will be assured that their decision to participate or not to participate in the study is completely voluntary and will not affect them in any way. All data will be anonymised prior to data analysis.

Links will be made with various NGOs and service providers prior to the commencement of the interviews to ensure efficient referral pathways. The research team anticipates a high number of referrals. As such, before the commencement of the interviews, various service providers were approached to ensure that the data collection team had a comprehensive referral directory. The referral directory has service providers in the fields of grief and loss, mental health, domestic violence, bullying and food insecurity. In addition, the participants will be provided with transport for their first appointment following referral.

Data analyses

Descriptive statistics will be used to explore data yielded from the questionnaire to explore a range of outcomes for children and adolescents at each of the study timepoints. A combination of analytical techniques will be utilised to explore different research questions inclusive of descriptive statistics (e.g., t-tests and chi-square tests), analyses of variance, multi-level regression modelling to account for potential confounders and repeat measures, and structural equation modelling (path analyses) with multiple outcomes to explore mediating and moderating factors in the relationship between predictor and outcome variables (identified apriori). Logistic regression modelling will be used to assess binary outcomes and linear regression modelling will be used to assess continuous outcomes.

Results will be disseminated via academic and policy publications, as well as national and international presentations inclusive of high-level meetings with technical experts. Findings will also be disseminated at a community level (e.g., through non-government organisations, community leaders, or local radio).

Discussion

This study aims to provide the first early insight into the experience of children following the death of a parent or primary caregiver, as a consequence of the COVID-19 pandemic, for children and adolescents aged 9-16 years living in the South Africa. The study will provide insight into their

For peer review only

needs, experiences and interventions that were provided, with an indication of those that were met, gaps in provision, unmet needs, and insights into future direction to optimise wellbeing in these children. Such data additionally aim to support planning for future pandemic responses. The use of a comparison group within this study will additionally, allow for the explorations regarding the possible differences in experiences among children and adolescents who had experienced caregiver loss compared to children and adolescents who had not experienced caregiver loss (e.g., experiences of lockdown). Such data will also allow for the exploration of cumulative risk among children and adolescents living in context of adversity (e.g., experiencing COVID-19 associated loss, poverty, and living in HIV-affected communities), and lend insight into the potential specific challenges related to the experience of caregiver loss in the wake of the COVID-19 pandemic.

Our research will generate evidence to inform further research, policy, and programming for at-risk orphans and vulnerable children, inclusive of mental health screening, and the exploration of modifiable risk and protective factors for health and wellbeing among this group. By producing knowledge about what works for orphans and vulnerable children it can be used to inform child health policies on a broad scale. We intend for our study to have wide applicability and have a clear path to dissemination. It is imperative to protect children and adolescents from the direct and indirect harms of the COVID-19 pandemic, both in the present and the long term. Data from this study provide a timely assessment of health and wellbeing in relation to caregiver loss with the aim of informing future responses to promote the potential of children and adolescents in South Africa and beyond.

Contributorship statement: LS, LC, MT, SS conceptualised the research study. All authors supported the development of the study, conceptualised research question, questionnaires, and methodologies. KSR, SDT and TM drafted the initial manuscript and led subsequent drafts. All authors read and provided feedback on manuscript iterations. All authors approved the final manuscript.

Competing interests: The authors declare no conflicts of interest.

Funding: UKRI GCRF Accelerating Achievement for Africa's Adolescents (Accelerate) Hub (Grant Ref: ES/S008101/1); Oak Foundation/GCRF "Accelerating Violence Prevention in Africa" [OFIL-20-057]; UNICEF ESARO; Wellspring Philanthropic Fund (grant no. 16204).

For peer review only

References

1. Hillis, S.D., et al., *Global minimum estimates of children affected by COVID-19-associated orphanhood and deaths of caregivers: a modelling study.* The Lancet, 2021. **398**(10298): p. 391-402.
2. Hillis, S., et al., *Orphanhood and caregiver loss among children based on new global excess COVID-19 death estimates.* JAMA pediatrics, 2022.
3. Joint United Nations Programme on HIV/AIDS. National AIDS Spending Assessment (NASA): Classification and Definitions. 2019: Geneva, Switzerland.
4. Raymond, J.M. and T.R. Zolnikov, *AIDS-affected orphans in sub-Saharan Africa: a scoping review on outcome differences in rural and urban environments.* AIDS and Behavior, 2018. **22**(10): p. 3429-3441.
5. Cluver, L.D., et al., *Persisting mental health problems among AIDS-orphaned children in South Africa.* Journal of Child Psychology and Psychiatry, 2012. **53**(4): p. 363-370.
6. Goodman, M.L., et al., *Child-street migration among HIV-affected families in Kenya: a mediation analysis from cross-sectional data.* AIDS care, 2016. **28**(sup2): p. 168-175.
7. Cluver, L., *Stigma, bullying, poverty and AIDS-orphanhood: Interactions mediating psychological problems for children in South Africa.* 2009.
8. Mkandawire, P., E. Tenkorang, and I.N. Luginaah, *Orphan status and time to first sex among adolescents in northern Malawi.* AIDS and Behavior, 2013. **17**(3): p. 939-950.
9. Skinner, D., et al., *A study of descriptive data for orphans and non-orphans on key criteria of economic vulnerability in two municipalities in South Africa.* curationis, 2013. **36**(1): p. 1-8.
10. Thomas, K.J., *Family contexts and schooling disruption among orphans in post-genocide Rwanda.* Population Research and Policy Review, 2010. **29**(6): p. 819-842.
11. Ardington, C. and M. Leibbrandt, *Orphanhood and schooling in South Africa: Trends in the vulnerability of orphans between 1993 and 2005.* Economic development and cultural change, 2010. **58**(3): p. 507-536.
12. Barenbaum, E. and T. Smith, *Social support as a protective factor for children impacted by HIV/AIDS across varying living environments in southern Africa.* AIDS care, 2016. **28**(sup2): p. 92-99.
13. Björkenstam, E., et al., *Childhood social adversity and risk of depressive symptoms in adolescence in a US national sample.* Journal of affective disorders, 2017. **212**: p. 56-63.
14. Carr, M., et al., *Self-harm and violent criminality linked with parental death during childhood.* Psychological medicine, 2020. **50**(7): p. 1224-1232.
15. Sherr, L., et al., *A systematic review on the meaning of the concept 'AIDS Orphan': confusion over definitions and implications for care.* AIDS care, 2008. **20**(5): p. 527-536.
16. Mahtab, S. and D. Coetzee, *Influence of HIV and other risk factors on tuberculosis.* South African Medical Journal, 2017. **107**(5): p. 428-438.
17. Super, G., *Violence and democracy in Khayelitsha, governing crime through the 'community'.* Stability: International Journal of Security and Development, 2015. **4**(1).
18. Seekings, J., *Economy, society and municipal services in Khayelitsha.* Report for the Commission of Inquiry into Allegations of Police Inefficiency in Khayelitsha and a breakdown in relations between the community and the police in Khayelitsha, Centre for Social Science Research, University of Cape Town, 2013.
19. City of Cape Town, *2011 Census – Khayelitsha Health District*. 2013.
20. Freeman, L. and C. McDonald, *Mapping Khayelitsha-The complexities of everyday policing in a high crime area.* South African Crime Quarterly, 2015. **53**: p. 27-37.
21. Sherr, L., et al., *Parenting, the other oldest profession in the world–a cross-sectional study of parenting and child outcomes in South Africa and Malawi.* Health psychology and behavioral medicine, 2017. **5**(1): p. 145-165.
22. Gordon, S., et al., *Maternal depressed mood and child development over the first five years of life in South Africa.* Journal of Affective Disorders, 2021. **294**: p. 346-356.

For peer review only

23. Tomlinson, M., et al., *First 1,000 days: enough for mothers but not for children? Long-term outcomes of an early intervention on maternal depressed mood and child cognitive development: follow-up of a randomised controlled trial.* Journal of Child Psychology and Psychiatry, 2022. **63**(3): p. 261-272.
24. Cluver, L.D., et al., *Violence prevention accelerators for children and adolescents in South Africa: A path analysis using two pooled cohorts.* PLoS medicine, 2020. **17**(11): p. e1003383.
25. Gittings, L., et al., *'Now my life is stuck!': Experiences of adolescents and young people during COVID-19 lockdown in South Africa.* Global public health, 2021. **16**(6): p. 947-963.
26. SSA, *Census 2001: Household Questionnaire. Pretoria: Statistics South Africa. Report No. 03–01-42*. 2001, Statistics South Africa Pretoria.
27. Marlow, M., et al., *Depression, anxiety, and psychological distress among caregivers of young children in rural Lesotho: Associations with food insecurity, household death and parenting stress.* SSM-Mental Health, 2022: p. 100167.
28. Knueppel, D., M. Demment, and L. Kaiser, *Validation of the household food insecurity access scale in rural Tanzania.* Public health nutrition, 2010. **13**(3): p. 360-367.
29. Kabalo, B.Y., et al., *Performance of an adapted household food insecurity access scale in measuring seasonality in household food insecurity in rural Ethiopia: a cohort analysis.* BMC nutrition, 2019. **5**(1): p. 1-10.
30. Du Toit, S., et al., *Accelerating progress towards improved mental health and healthy behaviours in adolescents living in adversity: findings from a longitudinal study in South Africa.* Psychology, Health & Medicine, 2022: p. 1-13.
31. Wright, M.G., *Findings from the indicators of poverty and social exclusion project: A profile of poverty using the socially perceived necessities approach.* 2008.
32. Pillay, U., et al., *South African social attitudes: changing times, diverse voices*. 2006: HSRC press.
33. Cluver, L., et al., *Child and adolescent suicide attempts, suicidal behavior, and adverse childhood experiences in South Africa: a prospective study.* Journal of Adolescent Health, 2015. **57**(1): p. 52-59.
34. Steventon Roberts, K.J., et al., *Adolescent mothers and their children affected by HIV—An exploration of maternal mental health, and child cognitive development.* Plos one, 2022. **17**(10): p. e0275805.
35. MT, J.B. and M.M. JM, *International classification of functioning, disability and health (ICF) 2001.* Revista espanola de salud publica, 2002. **76**(4): p. 271-279.
36. Pettifor, A.E., et al., *Young people's sexual health in South Africa: HIV prevalence and sexual behaviors from a nationally representative household survey.* Aids, 2005. **19**(14): p. 1525-1534.
37. Kovacs, M., *Children's depression inventory: Manual*. 1992: Multi-Health Systems North Tonawanda, NY.
38. Suliman, S., *Assessing post-traumatic responses among South African Adolescents: a comparison of different methods*. 2002, University of Cape Town.
39. Sherr, L., et al., *A foot in the door: A report on the Child Community Care stuyd evaluating the effect of Community Based Organisation support on child wellbeing in HIV affected communities*. 2017 Stellenbosch University & University College London London, UK p. 82.
40. Roberts, K.J., et al., *Adolescent motherhood and HIV in South Africa: Examining prevalence of common mental disorder.* AIDS and Behavior, 2022. **26**(4): p. 1197-1210.
41. Johnson, J.G., et al., *The patient health questionnaire for adolescents: validation of an instrument for the assessment of mental disorders among adolescent primary care patients.* Journal of Adolescent Health, 2002. **30**(3): p. 196-204.
42. Aggarwal, S., et al., *Evaluation of modified patient health questionnaire-9 teen in South African adolescents.* Indian Journal of Psychological Medicine, 2017. **39**(2): p. 143-145.

For peer review only

43. Rakshasa-Loots, A.M. and B. Laughton, *isiXhosa Translation of the Patient Health Questionnaire (PHQ-9): A Pilot Study of Psychometric Properties [Stage 1].* Frontiers in Psychiatry, 2022. **13**.
44. Chibanda, D., et al., *Validation of screening tools for depression and anxiety disorders in a primary care population with high HIV prevalence in Zimbabwe.* Journal of affective disorders, 2016. **198**: p. 50-55.
45. Spitzer, R.L., et al., *A brief measure for assessing generalized anxiety disorder: the GAD-7.* Archives of internal medicine, 2006. **166**(10): p. 1092-1097.
46. Kigozi, G., *Construct validity and reliability of the generalised anxiety disorder-7 scale in a sample of tuberculosis patients in the Free State Province, South Africa.* Southern African Journal of Infectious Diseases, 2021. **36**(1).
47. Adjorlolo, S., *Generalised anxiety disorder in adolescents in Ghana: Examination of the psychometric properties of the Generalised Anxiety Disorder-7 scale.* African Journal of Psychological Assessment, 2019. **1**(1).
48. Mughal, A.Y., et al., *A systematic review of validated screening tools for anxiety disorders and PTSD in low to middle income countries.* BMC psychiatry, 2020. **20**(1): p. 1-18.
49. Boyes, M.E., L.D. Cluver, and F. Gardner, *Psychometric properties of the child PTSD checklist in a community sample of South African children and adolescents.* PloS one, 2012. **7**(10): p. e46905.
50. Sheehan, D., D. Shytle, and K. Milo, *MINI KID: mini international neuropsychiatric interview for children and adolescents.* English Version, 2004. **4**: p. 0.
51. Sheehan, D., et al., *The validity of the Mini International Neuropsychiatric Interview (MINI) according to the SCID-P and its reliability.* European psychiatry, 1997. **12**(5): p. 232-241.
52. Lecrubier, Y., et al., *The Mini International Neuropsychiatric Interview (MINI). A short diagnostic structured interview: reliability and validity according to the CIDI.* European psychiatry, 1997. **12**(5): p. 224-231.
53. Burnett, P., et al., *Measuring core bereavement phenomena.* Psychological medicine, 1997. **27**(1): p. 49-57.
54. Thurman, T.R., et al., *Effect of a bereavement support group on female adolescents' psychological health: a randomised controlled trial in South Africa.* The Lancet Global Health, 2017. **5**(6): p. e604-e614.
55. Elgar, F.J., et al., *Development and validation of a short form of the Alabama Parenting Questionnaire.* Journal of Child and Family Studies, 2007. **16**(2): p. 243-259.
56. Cluver, L., et al., *A parenting programme to prevent abuse of adolescents in South Africa: study protocol for a randomised controlled trial.* Trials, 2016. **17**(1): p. 1-10.
57. Becker, S., *Young carers tasks and outcomes questionnaire.* Retrieved May, 2009. **7**: p. 2009.
58. Lane, T., L. Cluver, and D. Operario, *Young carers in South Africa: Tasks undertaken by children in households affected by HIV infection and other illness.* Vulnerable Children and Youth Studies, 2015. **10**(1): p. 55-66.
59. Achenbach, T., *Child behaviour checklists (CBCL/2-3 and CBCL/4-18), teacher report form (TRF) and youth self-report (YSR).* The handbook of psychiatric measures. 1st ed. Arlington, VA: The American Psychiatric Association, 2000.
60. Snider, L. and A. Dawes, *Psychosocial vulnerability and resilience measures for national-level monitoring of orphans and other vulnerable children: Recommendations for revision of the UNICEF Psychological Indicator.* Cape Town: UNICEF, 2006.
61. Amaya-Jackson, L., *Child's exposure to violence checklist.* Adapted from Richter's Things I've seen and heard, 1998.
62. Steventon Roberts, K., et al., *Risk factors for poor mental health among adolescent mothers in South Africa.* Psychology, Health & Medicine, 2022: p. 1-18.

For peer review only

63. Pantelic, M., et al., *HIV, violence, blame and shame: pathways of risk to internalized HIV stigma among South African adolescents living with HIV.* Journal of the international AIDS society, 2017. **20**(1): p. 21771.
64. Boyes, M.E. and L.D. Cluver, *Relationships between familial HIV/AIDS and symptoms of anxiety and depression: the mediating effect of bullying victimization in a prospective sample of South African children and adolescents.* Journal of youth and adolescence, 2015. **44**(4): p. 847-859.
65. Kieling, C., et al., *Child and adolescent mental health worldwide: evidence for action.* The Lancet, 2011. **378**(9801): p. 1515-1525.

For peer review only

STROBE Statement—Checklist of items that should be included in reports of ***cohort studies***

	Item No	Recommendation	Page No
**Title and abstract**	1	(*a*) Indicate the study's design with a commonly used term in the title or the abstract<br>(*b*) Provide in the abstract an informative and balanced summary of what was done and what was found	1,2
**Introduction**			
Background/rationale	2	Explain the scientific background and rationale for the investigation being reported	3
Objectives	3	State specific objectives, including any prespecified hypotheses	3
**Methods**			
Study design	4	Present key elements of study design early in the paper	4
Setting	5	Describe the setting, locations, and relevant dates, including periods of recruitment, exposure, follow-up, and data collection	3
Participants	6	(*a*) Give the eligibility criteria, and the sources and methods of selection of participants. Describe methods of follow-up<br>(*b*) For matched studies, give matching criteria and number of exposed and unexposed	4
Variables	7	Clearly define all outcomes, exposures, predictors, potential confounders, and effect modifiers. Give diagnostic criteria, if applicable	4
Data sources/ measurement	8*	For each variable of interest, give sources of data and details of methods of assessment (measurement). Describe comparability of assessment methods if there is more than one group	5-8
Bias	9	Describe any efforts to address potential sources of bias	9
Study size	10	Explain how the study size was arrived at	9
Quantitative variables	11	Explain how quantitative variables were handled in the analyses. If applicable, describe which groupings were chosen and why	9
Statistical methods	12	(*a*) Describe all statistical methods, including those used to control for confounding<br>(*b*) Describe any methods used to examine subgroups and interactions<br>(*c*) Explain how missing data were addressed<br>(*d*) If applicable, explain how loss to follow-up was addressed<br>(*e*) Describe any sensitivity analyses	9
**Results**			n/a
Participants	13*	(a) Report numbers of individuals at each stage of study—eg numbers potentially eligible, examined for eligibility, confirmed eligible, included in the study, completing follow-up, and analysed<br>(b) Give reasons for non-participation at each stage<br>(c) Consider use of a flow diagram	
Descriptive data	14*	(a) Give characteristics of study participants (eg demographic, clinical, social) and information on exposures and potential confounders<br>(b) Indicate number of participants with missing data for each variable of interest<br>(c) Summarise follow-up time (eg, average and total amount)	
Outcome data	15*	Report numbers of outcome events or summary measures over time	

For peer review only

Main results	16	(*a*) Give unadjusted estimates and, if applicable, confounder-adjusted estimates and their precision (eg, 95% confidence interval). Make clear which confounders were adjusted for and why they were included<br>(*b*) Report category boundaries when continuous variables were categorized<br>(*c*) If relevant, consider translating estimates of relative risk into absolute risk for a meaningful time period	
Other analyses	17	Report other analyses done—eg analyses of subgroups and interactions, and sensitivity analyses	
**Discussion**			
Key results	18	Summarise key results with reference to study objectives	
Limitations	19	Discuss limitations of the study, taking into account sources of potential bias or imprecision. Discuss both direction and magnitude of any potential bias	
Interpretation	20	Give a cautious overall interpretation of results considering objectives, limitations, multiplicity of analyses, results from similar studies, and other relevant evidence	
Generalisability	21	Discuss the generalisability (external validity) of the study results	
**Other information**			
Funding	22	Give the source of funding and the role of the funders for the present study and, if applicable, for the original study on which the present article is based	

*Give information separately for exposed and unexposed groups.

Note: An Explanation and Elaboration article discusses each checklist item and gives methodological background and published examples of transparent reporting. The STROBE checklist is best used in conjunction with this article (freely available on the Web sites of PLoS Medicine at http://www.plosmedicine.org/, Annals of Internal Medicine at http://www.annals.org/, and Epidemiology at http://www.epidem.com/). Information on the STROBE Initiative is available at http://www.strobe-statement.org.

For peer review only

For peer review only - http://bmjopen.bmj.com/site/about/guidelines.xhtml

BMJ Open

Protocol for the OCAY study: A cohort study of orphanhood and caregiver loss in the Covid-19 era to explore the impact on children and adolescents

Journal:	*BMJ Open*
Manuscript ID	bmjopen-2022-071023.R1
Article Type:	Protocol
Date Submitted by the Author:	02-May-2023
Complete List of Authors:	Steventon Roberts, Kathryn; University of Oxford, Department of Social Policy and Intervention ; University College London, Institute for Global Health<br>Du Toit, Stefani; Stellenbosch University<br>Mawoyo, Tatenda; Stellenbosch University<br>Tomlinson, Mark; University of Stellenbosch, Institute for Life Course Health Research<br>Cluver, Lucie; University of Oxford, Social Policy and Intervention<br>Skeen, Sarah; Stellenbosch University<br>Laurenzi, Christina; Stellenbosch University<br>Sherr, Lorraine; University College London
**Primary Subject Heading**:	Global health
Secondary Subject Heading:	Public health, Mental health
Keywords:	COVID-19, Public health < INFECTIOUS DISEASES, Community child health < PAEDIATRICS, PSYCHIATRY, PUBLIC HEALTH

SCHOLARONE™
Manuscripts

Protocol for the OCAY study: A cohort study of orphanhood and caregiver loss in the Covid-19 era to explore the impact on children and adolescents

Kathryn J. Steventon Roberts[1,2]*

Stefani Du Toit[3]*

Tatenda Mawoyo[3]*

Mark Tomlinson[3,4]

Lucie Cluver[1,5]

Sarah Skeen[3,6]

Christina A. Laurenzi[3]

Lorraine Sherr[2]

*Joint contributions

Corresponding author: Lorraine Sherr l.sherr@ucl.ac.uk

[1]Department for Social Policy and Intervention, University of Oxford, Oxford, UK

[2]Institute for Global Health, University College London, London, UK

[3]Institute for Life Course Health Research, Department of Global Health, Stellenbosch University, South Africa

[4]School of Nursing and Midwifery, Queens University, Belfast, UK

[5]Department of Psychiatry and Mental Health, University of Cape Town, Cape Town, South Africa

[6]Amsterdam Institute for Social Science Research, Faculty of Social and Behavioural Sciences, University of Amsterdam, Netherlands

For peer review only

Abstract

Introduction: Globally, no person has been untouched by the COVID-19 pandemic. Yet, little attention has been given to children and adolescents in policy, provision, and services. Moreover, there is a dearth of knowledge regarding the impact of COVID-19-associated orphanhood and caregiver loss on children. This study aims to provide early insights into the mental health and well-being of children and adolescents experiencing orphanhood or caregiver loss in South Africa.

Methods and analysis: Data will be drawn from a quantitative longitudinal study in Cape Town, South Africa. A sample of children and adolescents between the ages of 9 -18 years, experiencing parental or caregiver loss from COVID-19, will be recruited together with a comparison group of children in similar environments who did not experience loss. The study aims to recruit 500 children in both groups. Mental health and well-being among children will be explored through the use of validated and study specific measures. Participants will be interviewed at two time points, with follow-up data being collected 12–18 months after baseline. A combination of analytical techniques (including descriptive statistics, regression modelling and structural equation modelling) will be utilised to understand the experience and inform future policy and service provision.

Ethics and dissemination: This study received ethical approval from the Health Research Ethics Committee at Stellenbosch University (N 22/04/040). Results will be disseminated via academic and policy publications, as well as national and international presentations including high-level meetings with technical experts. Findings will also be disseminated at a community level via various platforms.

Strengths & Limitations

- This is the first empirical investigation of the impacts of COVID- 19 associated caregiver loss on the wellbeing of children and youth.
- A key strength of the study is the broad array of domains covered by the questionnaire used within this study and, the use of validated and standardised scales.
- The longitudinal nature of the study will strengthen investigations relating to the direction of any associations identified.
- At this stage data will only be drawn from the Western Cape province of South Africa, potentially restricting the generalisability of results beyond the region.

For peer review only

Background

The COVID-19 pandemic has had a dramatic impact on people across the world. The mortality rate from the virus has been high, with no country untouched, and notable deaths among parents and caregivers of children and adolescents – an estimated 10.5 million children and adolescents have experienced the death of a parent or caregiver [1, 2]. Yet, there has been little attention focused on the children and adolescents experiencing the death of parents and caregivers and the resulting consequences for their health and wellbeing. Orphanhood is defined as the death of one or both parents [3]. Orphanhood has previously been associated with an increased risk of poor mental health, violence and abuse exposure, unstable home environments, school disruption, early sexual debut, and poverty [4-11]. In many parts of the world, alternative caregivers, such as grandparents, are also key in providing essential care for children and adolescents. Due to the multigenerational nature of households, it is also important to consider caregiver loss as grandparents are often primary caregivers and were the most vulnerable to morbidity and mortality due to COVID-19 [1, 2, 12-14].

There needs to be a clear understanding and tracking of the nature of orphanhood and caregiver loss so that supportive provisions for children can be accurately tailored [15]. The occurrence of COVID-19 orphanhood and caregiver loss may well be layered onto pre-existing social systems where children already experience a multitude of social challenges and other forms of loss e.g., due to HIV or tuberculosis [16]. However, there may be elements of orphanhood and caregiver loss associated with COVID-19 that are new and yet undescribed and uncharted. Similarities from previous large-scale parental loss experiences such as HIV/AIDS, Ebola, war, and disaster needs to be understood while this new group of children and youth need to be linked to existing provision.

Undertaken within South Africa, the OCAY study is to the best of our knowledge, the first empirical study to explore the impact of orphanhood and caregiver loss on the wellbeing of children and adolescents. Children and adolescents who have experienced the loss of a caregiver due to COVID-19 and a comparison group of children and adolescents who have not experienced loss will be recruited to the study. There are five core objectives of the study: 1) to determine if mental health outcomes (positive/negative) differ between children and adolescents who have experienced a COVID-19 associated caregiver death, and children and adolescents who have not experienced a COVID-19 associated caregiver death; 2) to explore the impact of COVID-19 associated caregiver death on health and well-being outcomes of children, and service use (comparing those with and without a caregiver loss, and exploring impacts in relation sociodemographic factors e.g., experience of poverty); 3) to explore how mental health outcomes (positive/negative) among children and adolescents who have experienced orphanhood and caregiver loss change over time (utilising follow up data); 4) to examine associations between access to services (such as COVID-related grants, bereavement referrals) and outcomes for children and adolescents; 5) to compare the health and wellbeing status of children and adolescents who have lost a caregiver due to COVID-19 with children and adolescents who have lost a caregiver due to other causes (e.g., HIV/AIDS). Objectives will utilise both cross-sectional and longitudinal data to explore changes in experience over time. Based on previous literature, it is hypothesised that children and adolescents experiencing COVID-19 associated caregiver loss will experience worse health, well-being and service use outcomes compared to children who have not experienced caregiver loss.

Methods and analysis

Study setting

For peer review only

The study will predominantly take place in Khayelitsha, which is a under-resourced peri-urban settlement on the outskirts of Cape Town, South Africa. Khayelitsha is the third largest township in South Africa, with an estimated population of between 400 000 and 750 000 [17]. The majority of residents live in overcrowded informal housing with shared communal water taps and inadequate sanitation arrangements [17]. According to the 2011 Census, the average annual household income was R20 000 (approximately $1,200) [18]. There is a high unemployment rate in Khayelitsha, approximately 50% [19]. Khayelitsha experiences high levels of crime with inconsistent policing [20]. As existing studies forms part of our recruitment approach, participants residing in other parts of South Africa will also be considered for inclusion.

Participant recruitment

The study seeks to identify at least 500 children and adolescents aged between 9 – 18 years of age, who have been orphaned or lost a caregiver due to COVID-19 (defined as COVID-19 infection or likely COVID-19 infection). A similar proportion (n=500) of children and adolescents (9-18 years of age) who have not lost a parent or caregiver due to COVID-19 will be recruited as a comparison group. Various degrees of loss will be explored in the study (i.e., parental loss, other caregiver loss, no loss). To identify the sample, a mosaic of five strategies will be utilised: (1) *Existing studies* – as a starting point, participants included in previous studies conducted by the group's Primary Investigators (PI) and Co-primary Investigators (Co-PI) will be contacted to assess eligibility [21-25]. These studies include populations similar to the population of interest (e.g., age, sociodemographic characteristics) and are likely to include orphanhood-affected and non-affected participants (who have previously given consent to be contacted for future studies); (2) *Community links* – neighbourhoods within our study setting often have street committees, and these committees will be utilised to identify eligible participants. Alternatively, identification could take place by door-to-door recruitment; (3) *Schools* – following permission from the Western Cape Education Department (WCED), primary and high schools within the area will be approached for recruitment; (4) *Non-governmental organisations (NGOs)* – NGOs within our study setting, rendering services to children and adolescents, will be utilised; (5) *Snowball sampling* – participants recruited into the study will be provided with the opportunity to refer other potential participants.

Patient and Public Involvement

It was not appropriate or possible to involve patients or the public in the design, or conduct, or reporting, or dissemination plans of our research at the time of writing this protocol.

Study measures

All participants will be interviewed at two time points – baseline and 12-18 months later. Baseline data collection will take place between July 2022 - May 2023, with a second wave of data collection scheduled between July 2023 - May 2024. A study-specific inventory, adjusted to be age-appropriate, will be constructed. The components will use validated measures which have been used in five previous studies undertaken with children and adolescents within South Africa (the Young Carers study, the Mzansti Wakho study, the HEY BABY study, the Child Community Care Study, and the Adolescent Bereavement Initiative). Given the broad extent of the impact of COVID-19 and orphanhood on the lives of the children and adolescents, measures (for both those affected by orphanhood, and the non-affected group) will explore multiple aspects of life including: (1) demographic and social circumstance factors; (2) mental health (including depression, anxiety, posttraumatic stress and suicidality) and wellbeing; (3) COVID-19 experience; (4) bereavement experience; (5) care and subsequent care experience; (6) experience of support & provision (7)

For peer review only

health experience; (8) education and school experience; (9) behavioural risk; (10) violence exposure, stigma and bullying experience; and (11) parenting and caregiving experience. Data relating to the period between caregiver loss and baseline data collection will also be obtained to ensure that time since loss is accounted for within analyses. Measures will be piloted and refined/adapted based on participant feedback. Piloting will be undertaken with 10-15 participants who have been identified as eligible for the study within the greater Khayelitsha area. Study measures will remain similar across both waves, however minor amendments may be made based on feedback (e.g., from data collectors and/or the study team) from baseline data collection. Measures that remain static over time will not be included within follow-up data collection. See Table 1 for an overview of measures to be utilised within the study.

For peer review only

Table 1. Study measures for inclusion within the OCAY study

Domain	Measures	Example items & response categories	Examples of previous use and/or validation within LMIC
**Demographic & social circumstance factors**			
Demographic information	Child age, biological sex, gender, and basic demographic information will be obtained from items utilizing the South African Census [26].	-	-
Anthropometric measurements	Height and weight will be obtained to assess physical development	-	-
Household food security	Study specific items adapted from the household food insecurity access scale (10 items) which measures food security in the previous 30 days	E.g., Did you worry that your household would not have enough food?<br><br>Response: Never/Rarely/Sometimes/Often	Marlow et al (South Africa)[27]<br><br>Knueppel et al (Tanzania) [28]<br><br>Kabalo et al (Ethiopia) [29]
Socioeconomic status	Do you have running water? Do you have electricity? Do you have a flush toilet?	E.g., Do you have running water at your house?<br><br>Response: Yes/No	Du Toit et al (South Africa)[30]
	Socioeconomic status will also be measured by measuring access to the top 8 socially-perceived necessities for children, as identified by the Centre for South African Social Policy in the 'Indicators of poverty and social exclusion project' [31] and endorsed by over 80% of the South African population in a nationally-representative survey (the South African Social Attitudes Survey 2006) [32]. These include items such as 'enough clothes to keep you warm and dry'.	Please tick all of the things that you can afford at home….<br><br>Response: E.g., 3 meals a day, school fees	Cluver et al (South Africa)[33]<br><br>Steventon Roberts et al (South Africa)[34]
	Cash grant receipt	E.g., Are you or anyone in your household receiving any grants?	-
	Household employment	E.g., How many people in your household are employed?	-
Household information	Study specific questions on household arrangements & living circumstances	E.g., What type of house do you live in?<br><br>Response: E.g., House made of bricks on a separate yard/block of flats	-
**Health**			
COVID-19	Study specific questions on experience of COVID-19 including caregiver death, COVID-19 infection within the household, experience of lockdown and presence of long COVID	E.g., Were you or anyone in your household infected with COVID-19?<br><br>Response: Yes/No	-
HIV	Study specific questions on household HIV Status, testing, status, and treatment	E.g., Is anyone in your household living with HIV?<br><br>Response: Yes/No/Don't know/Decline to answer	-
Overall health status	An adapted subscale from the International Classification of Functioning Disability and Health (3-items) will be utilized to assess self-reported health within the previous 12 months[35]	E.g., In the last 12 months, did you have difficulty remembering things or following a story or conversation?<br><br>Response: No, no difficulty at all/Yes, some difficulty/Yes, a lot of difficulty/Cannot do it at all	-
Sexual and Reproductive Health	Study specific questions adapted from the National Survey and risk behaviour amongst South Africans [36] will be used to explores sexual and reproductive health. Items focus on sexual activity, age of sexual debut, contraception use, transactional sex, substance use during sexual activity, pregnancy & sexual abuse	E.g., Are you currently in a sexual and/or romantic relationship or have you ever been in a sexual and/or romantic relationship?<br><br>Response: Yes/No	-
Health service utilisation	Study specific questions regarding what health services have been accessed by the participant in the previous 12 months	E.g., Which of the following health services have you accessed in the last 12 months?	-
**Mental health**			
Depressive symptoms	The Child Depression Inventory Short Form (10 items; CDI-SF) [37] was used to assess depressive symptomology within the previous two weeks.	E.g., Select the statement which best describes how you have felt in the past 2 weeks.<br><br>Response: Nothing will ever work out for me/ I am not sure if things will work out for me/ Things will work out for me OK	Suliman et al (South Africa) [38]<br><br>Cluver et al (South Africa) [33]<br><br>Sherr et al (South Africa, Malawi, Zambia) [39]<br><br>Roberts et al (South Africa) [40]
	The Patient Health Questionnaire – 9 (9 items; PHQ-9) [41] was used to assess depressive symptomology within the previous two weeks.	E.g., Have you felt down, depressed, irritable, or hopeless?<br><br>Response: Not at all/Several days/More than half the days/Nearly every day	Aggarwal et al (South Africa) [42]<br><br>Rakshasa-Loots et al (South Africa) [43]<br><br>Chibanda et al (Zimbabwe) [44]
Anxiety symptoms	The Generalized Anxiety Disorder-7 (7 items; GAD-7) [45] was used to assess anxiety symptoms in the previous two weeks	E.g., Have you felt nervous, anxious or on edge?<br><br>Response: Not at all/Several days/More than half the days/Nearly every day	Kigozi et al (South Africa) [46]<br><br>Adjorlolo et al (Ghana) [47]<br><br>Roberts et al (South Africa) [40]<br><br>Chibanda et al (Zimbabwe) [44]<br><br>Mughal et al (LMIC) [48]
Posttraumatic stress symptoms	A single item informed by the Child PTSD checklist was used to explore posttraumatic stress symptoms among those children and adolescents' who had lost a caregiver	How stressed have you been since the death of your caregiver?<br><br>Response: Very/A little/No more than usual	Boyes et al (South Africa) [49]<br><br>Roberts et al (South Africa) [40]
Suicidality and self-harm	Mini International Psychiatric Interview for Children and Adolescents Suicidality (5 items; MINI-Kid) [50] was used to assess suicidality and self-harm behaviours in the previous month	E.g., In the past month did you wish you were dead?<br><br>Response: Yes/No	Sheehan et al & Lecrubier et al (Global) [51, 52].<br><br>Cluver et al (South Africa) [33]<br><br>Roberts et al (South Africa) [40]
Self-esteem	The Rosenberg Self-Esteem Scale (10 items) [39] was used as an assessment of child and adolesent self-esteem	E.g., I feel that I have a number of good qualities	Sherr et al (South Africa, Malawi, Zambia) [39]

For peer review only

For peer review only - http://bmjopen.bmj.com/site/about/guidelines.xhtml

		Response: Strongly agree/Agree/Disagree/Strongly Disagree	
Access to mental health services	A single study specific item to assess whether children and adolescents have access to mental health services	Do you have access to mental health services?<br><br>Response: Yes, needed and accessed/Yes, but didn't need so didn't access/No, needed but couldn't access /No, didn't need and didn't access	-
**Bereavement experience**			
Grief	The Core Bereavement Items – Grief sub-scale (5 items) will be used to assess grief intensity [53]	E.g., Do reminders of X such as photos, situations, music, places etc. cause you to feel longing for X?<br><br>Response: A lot of the time/Quite a bit of the time/A little bit of the time/Never	Thurman et al (South Africa) [54]
**Parenting & caregiving experience**			
Parenting experience	The Alabama Parenting Questionnaire (16 items) will be used to identify experience of positive parenting, caregiver supervision, experience of caregiver discipline within the previous two months[55]	E.g., Thinking about the person who looks after you the most, your parent or caregiver says that you have done something well...<br><br>Response: Never/Rarely/Sometimes/Often/Always	Cluver et al (South Africa)[56]<br><br>Elgar et al (Global) [55]
Experience of caregiving and responsibility for household tasks	The Young Carers Tasks & Outcomes Questionnaire (23 items [57] was adapted to assess children and adolescents experiences of responsibility for household tasks	E.g., In the past week has you washed other people's clothes?<br><br>Response: Yes/No<br><br>How many days a week do you do this?	Lane et al (South Africa) [58]
**Support interventions & provision**			
Organisational support	Study specific questions on support from organisations e.g., support from social services, healthcare workers	E.g., Do you have access to an organisation that provides you with any support services?<br><br>Response: Yes/No	-
Community provision	Study specific questions on community provisions	E.g., Are you a member of any youth and/or health organisations, political or activist groups?	-
**Education & school experiences**			
Education	Study specific questions on school enrolment, being in the correct class for age, educational attainment, education aspirations and school dropout.	E.g., Are you currently enrolled in school?<br><br>Response: Yes/No	-
**Behavioural risk**			
Behavioural problems	The Child Behaviour Checklist: Rule-breaking subscale (17 items) was used to assesses behavioural problems in the previous 6 months[59]	E.g., I drink alcohol to have fun, without my caregivers knowing or approving<br><br>Response: Not true/Somewhat or sometimes true/Very true or often true	Sherr et al (South Africa, Malawi, Zambia) [39]
**Violence exposure, stigma & bullying**			
Violence exposure	Study specific questions adapted from the UNICEF measures for national level monitoring of orphans and vulnerable children [60] and the Parent-Child Conflict Tactics Scales were used to assess household, domestic, and school violence	E.g., How often has anyone in your family or living in your home or someone at school used a stick, belt, or other hard item to hit you?<br><br>Response: Weekly/Monthly/At least once a year/Has happened but not in the last year/Never	-
Community violence	The child exposure to community violence checklist [61] will be used to identify children and adolescents' exposure to community violence	E.g., Have you ever been hot or attacked outside your home?<br><br>Response: Yes, more than a year ago/Yes, in the last year/Never	Sherr et al (South Africa, Malawi, Zambia) [39]<br><br>Steventon Roberts et al (South Africa) [62]
Stigma	Study specific questions focusing on COVID-19 related stigma	E.g., I have been treated badly because I lost a caregiver to COVID-19<br><br>Response: Not at all/Sometimes/All of the time	-
Bullying	The Social and Health Assessment Peer Victimisation Scale (12 items) was used to examine experiences of bullying in the last 6 months[63]	E.g., In the past 6 months other kids have called me names or swore at me...<br><br>Response: Not at all/Once/2-3times/4 or more times	Boyes et al (South Africa) [64]<br><br>Sherr et al (South Africa, Malawi, Zambia) [39]

Note. Low- and middle-income countries (LMIC)

For peer review only

Power calculation

Power calculations were based on the core mental health measures within the study (depressive/anxiety symptoms). Multiple power calculations have informed this estimate. Previous studies identifying approximately 10% of children and adolescents experience poor mental health [65]. Stratification modelling data based on the methodologies used in Hillis et al. 2022,[2] identified that caregiver loss within South Africa due to COVID-19 has impacted approximately 211 000 children (estimate obtained November 2022; COVID-19 Orphanhood Calculator). Based on this data, a confidence level of 95% and a 2% margin of error the sample size required to detect meaningful difference in the proportion of poor mental health symptomology reported between the sample and general population estimates (Chi-square test) is 861. A second power calculation was undertaken to account for group differences (COVID-19 caregiver loss vs. no COVID-19 caregiver loss) in the core mental health measures (prevalence). Based on identifying a prevalence of 5% poor mental health symptoms (depression/anxiety) among the control group (no COVID-19 associated orphanhood) and 10% prevalence within the population of interest (COVID-19 associated orphanhood), an alpha of 0.05, and 90% power the number of participants required to detect a meaningful difference between groups is 671 (COVID-19 associated orphanhood) vs. 503 (no COVID-19 associated orphanhood). Based on these estimates, and to account for attrition in planned follow-up rounds of data collection, a planned sample of 1,000 children including the number of children and adolescents experiencing COVID-19 associated orphanhood or caregiver loss n=500, and the comparison group of children and adolescents experiencing no COVID-19 associated orphanhood n= 500.

Data collection procedures

Data will be collected by experienced, trained, and supervised data collectors. Data collectors, with extensive prior experience in quantitative research in the study site, will undergo a two-week refresher training workshop in general principles of research ethics, referral procedures, management of difficult situations, obtaining informed consent, and how to administer the questionnaire. Data collectors will be fluent in English and isiXhosa (the vernacular language of Khayelitsha residents), with prior experience working with vulnerable youth and families in these settings. Assessments will take place at a research centre in Khayelitsha. In the instances where participants are unable to travel to the research centre, assessments will be conducted in a private space at the participant's home, school, or NGO. In the instances where participants reside in other parts of South Africa, we will conduct telephonic assessments. Participants will be provided with transportation to and from the venue where assessments are taking place. Prior to starting the interview, caregivers of children under the age of 18 will provide written consent for the participation of their child, and the child will provide informed assent. Participants who are 18 years of age will provide their own informed consent. All measures will be translated into isiXhosa and back-translated into English to ensure the use of accurate and quality translations. Interviews are expected to last between 60-90 minutes. During assessment, participants will be provided with breaks during which they will be served refreshments. All participants are provided with a reimbursement for their time, inconvenience, and expense in the form of a shopping voucher, and these reimbursements will be provided at each time point. As a means of quality control, the assessments will be audio-recorded, with the permission of the participant. These audio-recordings are reviewed by supervisory staff on a regular basis to ensure consistency in administration between data collectors. Upon completion of the assessments, participants will be provided with a further opportunity to ask any questions or express any concerns.

For peer review only

The second wave of data collection will follow a similar format to the first wave of data collection. As with the first wave, data collectors will undergo a refresher training workshop. Participants will be re-contacted by the study team. Retention will be a core priority within the second round of data collection. The study team are known to the local community. Participants will be contacted on numerous occasions by the data collection team. The core study team have an excellent track-record of high retention rates within previous studies among similar populations (e.g., the Child Community Care study – 89%, the Mzantsi Wakho study – 95%).

Data analyses

Descriptive statistics will be used to explore data yielded from the questionnaire to explore a range of outcomes for children and adolescents at each of the study timepoints. Explorations will be based on study objectives. For illustrative purposes and to guide possible analyses, Figure 1 presents directed acyclic graphs for each of the study objectives (see Figure 1). A combination of analytical techniques will be utilised to explore different research questions inclusive of descriptive statistics (e.g., t-tests and chi-square tests), analyses of variance, multi-level regression modelling to account for potential confounders and repeat measures, and structural equation modelling (path analyses) with multiple outcomes to explore mediating and moderating factors in the relationship between predictor and outcome variables (identified apriori). Logistic regression modelling will be used to assess binary outcomes and linear regression modelling will be used to assess continuous outcomes. If possible, the above analytical techniques will also be used to explore additional data from existing sources to compare the health and wellbeing outcomes of children and adolescents experiencing COVID-19 associated loss with other groups e.g., children and adolescents experiencing caregiver loss due to HIV/AIDS.

Ethics and dissemination

This study has received ethical approval from the Health Research Ethics Committee at Stellenbosch University (N22/04/040). This research is designed with child protection principles in mind, and the impact of the questionnaire is a foremost concern. The study will follow ethical procedures used by the researchers in other similar studies working with similar at-risk groups. The questionnaire will be designed to be child-friendly. All consent procedures will be conducted prior the commencement of the assessment. Given the young age of some participants and the sensitive nature of the project, all screening and consent processes will be conducted in the homes of the prospective participants. Hopefully, conducting screening and consent procedures in the comfort of the participants' homes will build trust and enhance rapport with both the participants and the caregivers. Caregivers will be welcome to accompany their child to the interview, however, if this is not possible, written consent will be obtained prior to the scheduled appointment and permission will be sought to transport the child to and from the research centre or interview venue. Data collectors will explain the interview and assessment procedures to each child, and they will sign an assent form. Caregivers will sign a consent form to provide permission for their child to participate in the study. All participants will be assured that their decision to participate or not to participate in the study is completely voluntary and will not affect them in any way. All data will be anonymised prior to data analysis.

Links will be made with various NGOs and service providers prior to the commencement of the interviews to ensure efficient referral pathways. The research team anticipates a high number of referrals. As such, before the commencement of the interviews, various service providers were

For peer review only

approached to ensure that the data collection team had a comprehensive referral directory. The referral directory has service providers in the fields of grief and loss, mental health, domestic violence, bullying and food insecurity. In addition, the participants will be provided with transport for their first appointment following referral.

Results will be disseminated via academic and policy publications, as well as national and international presentations inclusive of high-level meetings with technical experts. Findings will also be disseminated at a community level (e.g., through specially-convened community stakeholder meetings, non-government organisations, community leaders, or local radio).

This study aims to provide early data on the experiences of children and adolescents ages 9-18 following the death of a parent or caregiver as a consequence of the COVID-19 pandemic in South Africa. The study will provide insight into their needs, experiences and interventions that were provided, with an indication of gaps in provision, unmet needs, and areas where future support could be targeted to optimise well-being in these children. Such data additionally aim to support planning for future pandemic responses. The use of a comparison group within this study will additionally allow for the explorations regarding the possible differences in experiences among children and adolescents who had experienced caregiver loss compared to children and adolescents who had not experienced caregiver loss (e.g., experiences of lockdown). Similarly, this data will allow for the exploration of cumulative risk among children and adolescents living in contexts of adversity (e.g., experiencing COVID-19 associated loss, poverty, and living in HIV-affected communities), and help to identify the potential specific challenges related to the experience of caregiver loss in the wake of the COVID-19 pandemic.

Our research will generate evidence to inform further research, policy, and programming for at-risk orphans and vulnerable children, including mental health screening and modifiable risk and protective factors for health and well-being among this group. Enhanced knowledge about what works for orphans and vulnerable children can be used to inform child health policies on a broad scale. We intend for our study to have wide applicability and have a clear path to dissemination. It is imperative to protect children and adolescents from the direct and indirect harms of the COVID-19 pandemic, both in the present and the long term. Data from this study provide a timely assessment of health and wellbeing in relation to caregiver loss with the aim of informing future responses to promote the potential of children and adolescents in South Africa and beyond.

Author contributions: LS, LC, MT, SS conceptualised the research study. All authors supported the development of the study, conceptualised research question, questionnaires, and methodologies. KJSR, SDT, TM and CAL drafted the initial manuscript and led subsequent drafts. All authors read and provided feedback on manuscript iterations. All authors approved the final manuscript.

Funding statement: UKRI Global Challenges Research Fund (GCRF) Accelerating Achievement for Africa's Adolescents (Accelerate) Hub (Grant Ref: ES/S008101/1); Oak Foundation/GCRF "Accelerating Violence Prevention in Africa" [OFIL-20-057]; UNICEF ESARO; Wellspring Philanthropic Fund (grant no. 16204).

Competing interests statement: The authors declare no conflicts of interest.

For peer review only

References

1. Hillis, S.D., et al., *Global minimum estimates of children affected by COVID-19-associated orphanhood and deaths of caregivers: a modelling study.* The Lancet, 2021. **398**(10298): p. 391-402.
2. Hillis, S., et al., *Orphanhood and caregiver loss among children based on new global excess COVID-19 death estimates.* JAMA pediatrics, 2022.
3. Joint United Nations Programme on HIV/AIDS. National AIDS Spending Assessment (NASA): Classification and Definitions. 2019: Geneva, Switzerland.
4. Raymond, J.M. and T.R. Zolnikov, *AIDS-affected orphans in sub-Saharan Africa: a scoping review on outcome differences in rural and urban environments.* AIDS and Behavior, 2018. **22**(10): p. 3429-3441.
5. Cluver, L.D., et al., *Persisting mental health problems among AIDS-orphaned children in South Africa.* Journal of Child Psychology and Psychiatry, 2012. **53**(4): p. 363-370.
6. Goodman, M.L., et al., *Child-street migration among HIV-affected families in Kenya: a mediation analysis from cross-sectional data.* AIDS care, 2016. **28**(sup2): p. 168-175.
7. Cluver, L., *Stigma, bullying, poverty and AIDS-orphanhood: Interactions mediating psychological problems for children in South Africa.* 2009.
8. Mkandawire, P., E. Tenkorang, and I.N. Luginaah, *Orphan status and time to first sex among adolescents in northern Malawi.* AIDS and Behavior, 2013. **17**(3): p. 939-950.
9. Skinner, D., et al., *A study of descriptive data for orphans and non-orphans on key criteria of economic vulnerability in two municipalities in South Africa.* curationis, 2013. **36**(1): p. 1-8.
10. Thomas, K.J., *Family contexts and schooling disruption among orphans in post-genocide Rwanda.* Population Research and Policy Review, 2010. **29**(6): p. 819-842.
11. Ardington, C. and M. Leibbrandt, *Orphanhood and schooling in South Africa: Trends in the vulnerability of orphans between 1993 and 2005.* Economic development and cultural change, 2010. **58**(3): p. 507-536.
12. Barenbaum, E. and T. Smith, *Social support as a protective factor for children impacted by HIV/AIDS across varying living environments in southern Africa.* AIDS care, 2016. **28**(sup2): p. 92-99.
13. Björkenstam, E., et al., *Childhood social adversity and risk of depressive symptoms in adolescence in a US national sample.* Journal of affective disorders, 2017. **212**: p. 56-63.
14. Carr, M., et al., *Self-harm and violent criminality linked with parental death during childhood.* Psychological medicine, 2020. **50**(7): p. 1224-1232.
15. Sherr, L., et al., *A systematic review on the meaning of the concept 'AIDS Orphan': confusion over definitions and implications for care.* AIDS care, 2008. **20**(5): p. 527-536.
16. Mahtab, S. and D. Coetzee, *Influence of HIV and other risk factors on tuberculosis.* South African Medical Journal, 2017. **107**(5): p. 428-438.
17. Super, G., *Violence and democracy in Khayelitsha, governing crime through the 'community'.* Stability: International Journal of Security and Development, 2015. **4**(1).
18. Seekings, J., *Economy, society and municipal services in Khayelitsha.* Report for the Commission of Inquiry into Allegations of Police Inefficiency in Khayelitsha and a breakdown in relations between the community and the police in Khayelitsha, Centre for Social Science Research, University of Cape Town, 2013.
19. City of Cape Town, *2011 Census – Khayelitsha Health District*. 2013.
20. Freeman, L. and C. McDonald, *Mapping Khayelitsha-The complexities of everyday policing in a high crime area.* South African Crime Quarterly, 2015. **53**: p. 27-37.
21. Sherr, L., et al., *Parenting, the other oldest profession in the world–a cross-sectional study of parenting and child outcomes in South Africa and Malawi.* Health psychology and behavioral medicine, 2017. **5**(1): p. 145-165.
22. Gordon, S., et al., *Maternal depressed mood and child development over the first five years of life in South Africa.* Journal of Affective Disorders, 2021. **294**: p. 346-356.

For peer review only

23. Tomlinson, M., et al., *First 1,000 days: enough for mothers but not for children? Long-term outcomes of an early intervention on maternal depressed mood and child cognitive development: follow-up of a randomised controlled trial.* Journal of Child Psychology and Psychiatry, 2022. **63**(3): p. 261-272.
24. Cluver, L.D., et al., *Violence prevention accelerators for children and adolescents in South Africa: A path analysis using two pooled cohorts.* PLoS medicine, 2020. **17**(11): p. e1003383.
25. Gittings, L., et al., *'Now my life is stuck!': Experiences of adolescents and young people during COVID-19 lockdown in South Africa.* Global public health, 2021. **16**(6): p. 947-963.
26. SSA, *Census 2001: Household Questionnaire. Pretoria: Statistics South Africa. Report No. 03–01-42*. 2001, Statistics South Africa Pretoria.
27. Marlow, M., et al., *Depression, anxiety, and psychological distress among caregivers of young children in rural Lesotho: Associations with food insecurity, household death and parenting stress.* SSM-Mental Health, 2022: p. 100167.
28. Knueppel, D., M. Demment, and L. Kaiser, *Validation of the household food insecurity access scale in rural Tanzania.* Public health nutrition, 2010. **13**(3): p. 360-367.
29. Kabalo, B.Y., et al., *Performance of an adapted household food insecurity access scale in measuring seasonality in household food insecurity in rural Ethiopia: a cohort analysis.* BMC nutrition, 2019. **5**(1): p. 1-10.
30. Du Toit, S., et al., *Accelerating progress towards improved mental health and healthy behaviours in adolescents living in adversity: findings from a longitudinal study in South Africa.* Psychology, Health & Medicine, 2022: p. 1-13.
31. Wright, M.G., *Findings from the indicators of poverty and social exclusion project: A profile of poverty using the socially perceived necessities approach.* 2008.
32. Pillay, U., et al., *South African social attitudes: changing times, diverse voices*. 2006: HSRC press.
33. Cluver, L., et al., *Child and adolescent suicide attempts, suicidal behavior, and adverse childhood experiences in South Africa: a prospective study.* Journal of Adolescent Health, 2015. **57**(1): p. 52-59.
34. Steventon Roberts, K.J., et al., *Adolescent mothers and their children affected by HIV—An exploration of maternal mental health, and child cognitive development.* Plos one, 2022. **17**(10): p. e0275805.
35. MT, J.B. and M.M. JM, *International classification of functioning, disability and health (ICF) 2001.* Revista espanola de salud publica, 2002. **76**(4): p. 271-279.
36. Pettifor, A.E., et al., *Young people's sexual health in South Africa: HIV prevalence and sexual behaviors from a nationally representative household survey.* Aids, 2005. **19**(14): p. 1525-1534.
37. Kovacs, M., *Children's depression inventory: Manual*. 1992: Multi-Health Systems North Tonawanda, NY.
38. Suliman, S., *Assessing post-traumatic responses among South African Adolescents: a comparison of different methods*. 2002, University of Cape Town.
39. Sherr, L., et al., *A foot in the door: A report on the Child Community Care stuyd evaluating the effect of Community Based Organisation support on child wellbeing in HIV affected communities*. 2017 Stellenbosch University & University College London London, UK p. 82.
40. Roberts, K.J., et al., *Adolescent motherhood and HIV in South Africa: Examining prevalence of common mental disorder.* AIDS and Behavior, 2022. **26**(4): p. 1197-1210.
41. Johnson, J.G., et al., *The patient health questionnaire for adolescents: validation of an instrument for the assessment of mental disorders among adolescent primary care patients.* Journal of Adolescent Health, 2002. **30**(3): p. 196-204.
42. Aggarwal, S., et al., *Evaluation of modified patient health questionnaire-9 teen in South African adolescents.* Indian Journal of Psychological Medicine, 2017. **39**(2): p. 143-145.

For peer review only

43. Rakshasa-Loots, A.M. and B. Laughton, *isiXhosa Translation of the Patient Health Questionnaire (PHQ-9): A Pilot Study of Psychometric Properties [Stage 1].* Frontiers in Psychiatry, 2022. **13**.
44. Chibanda, D., et al., *Validation of screening tools for depression and anxiety disorders in a primary care population with high HIV prevalence in Zimbabwe.* Journal of affective disorders, 2016. **198**: p. 50-55.
45. Spitzer, R.L., et al., *A brief measure for assessing generalized anxiety disorder: the GAD-7.* Archives of internal medicine, 2006. **166**(10): p. 1092-1097.
46. Kigozi, G., *Construct validity and reliability of the generalised anxiety disorder-7 scale in a sample of tuberculosis patients in the Free State Province, South Africa.* Southern African Journal of Infectious Diseases, 2021. **36**(1).
47. Adjorlolo, S., *Generalised anxiety disorder in adolescents in Ghana: Examination of the psychometric properties of the Generalised Anxiety Disorder-7 scale.* African Journal of Psychological Assessment, 2019. **1**(1).
48. Mughal, A.Y., et al., *A systematic review of validated screening tools for anxiety disorders and PTSD in low to middle income countries.* BMC psychiatry, 2020. **20**(1): p. 1-18.
49. Boyes, M.E., L.D. Cluver, and F. Gardner, *Psychometric properties of the child PTSD checklist in a community sample of South African children and adolescents.* PloS one, 2012. **7**(10): p. e46905.
50. Sheehan, D., D. Shytle, and K. Milo, *MINI KID: mini international neuropsychiatric interview for children and adolescents.* English Version, 2004. **4**: p. 0.
51. Sheehan, D., et al., *The validity of the Mini International Neuropsychiatric Interview (MINI) according to the SCID-P and its reliability.* European psychiatry, 1997. **12**(5): p. 232-241.
52. Lecrubier, Y., et al., *The Mini International Neuropsychiatric Interview (MINI). A short diagnostic structured interview: reliability and validity according to the CIDI.* European psychiatry, 1997. **12**(5): p. 224-231.
53. Burnett, P., et al., *Measuring core bereavement phenomena.* Psychological medicine, 1997. **27**(1): p. 49-57.
54. Thurman, T.R., et al., *Effect of a bereavement support group on female adolescents' psychological health: a randomised controlled trial in South Africa.* The Lancet Global Health, 2017. **5**(6): p. e604-e614.
55. Elgar, F.J., et al., *Development and validation of a short form of the Alabama Parenting Questionnaire.* Journal of Child and Family Studies, 2007. **16**(2): p. 243-259.
56. Cluver, L., et al., *A parenting programme to prevent abuse of adolescents in South Africa: study protocol for a randomised controlled trial.* Trials, 2016. **17**(1): p. 1-10.
57. Becker, S., *Young carers tasks and outcomes questionnaire.* Retrieved May, 2009. **7**: p. 2009.
58. Lane, T., L. Cluver, and D. Operario, *Young carers in South Africa: Tasks undertaken by children in households affected by HIV infection and other illness.* Vulnerable Children and Youth Studies, 2015. **10**(1): p. 55-66.
59. Achenbach, T., *Child behaviour checklists (CBCL/2-3 and CBCL/4-18), teacher report form (TRF) and youth self-report (YSR).* The handbook of psychiatric measures. 1st ed. Arlington, VA: The American Psychiatric Association, 2000.
60. Snider, L. and A. Dawes, *Psychosocial vulnerability and resilience measures for national-level monitoring of orphans and other vulnerable children: Recommendations for revision of the UNICEF Psychological Indicator.* Cape Town: UNICEF, 2006.
61. Amaya-Jackson, L., *Child's exposure to violence checklist.* Adapted from Richter's Things I've seen and heard, 1998.
62. Steventon Roberts, K., et al., *Risk factors for poor mental health among adolescent mothers in South Africa.* Psychology, Health & Medicine, 2022: p. 1-18.

For peer review only

63. Pantelic, M., et al., *HIV, violence, blame and shame: pathways of risk to internalized HIV stigma among South African adolescents living with HIV.* Journal of the international AIDS society, 2017. **20**(1): p. 21771.
64. Boyes, M.E. and L.D. Cluver, *Relationships between familial HIV/AIDS and symptoms of anxiety and depression: the mediating effect of bullying victimization in a prospective sample of South African children and adolescents.* Journal of youth and adolescence, 2015. **44**(4): p. 847-859.
65. Kieling, C., et al., *Child and adolescent mental health worldwide: evidence for action.* The Lancet, 2011. **378**(9801): p. 1515-1525.

Figure Legend: Note: Objective 1: to determine if mental health outcomes (positive/negative) differ between children and adolescents who have experienced a COVID-19 associated caregiver death, and children and adolescents who have not experienced a COVID-19 associated caregiver death; Objective 2) to explore the impact of COVID-19 associated caregiver death on health and wellbeing outcomes of children, and service use (comparing those with and without a caregiver loss, and exploring impacts in relation sociodemographic factors e.g., experience of poverty); Objective 3) to explore how mental health outcomes (positive/negative) among children and adolescents who have experienced orphanhood and caregiver loss change over time (utilising follow up data); Objective 4) to examine associations between access to services (such as COVID-related grants, bereavement referrals) and outcomes for children and adolescents; Objective 5) to compare the health and wellbeing status of children and adolescents who have lost a caregiver due to COVID-19 with children and adolescents who have lost a caregiver due to other causes (e.g., HIV/AIDS).

Figure 1. Directed Acyclic Graphs illustrative of the OCAY study objectives (for illustrative purposes only)

For peer review only

For peer review only

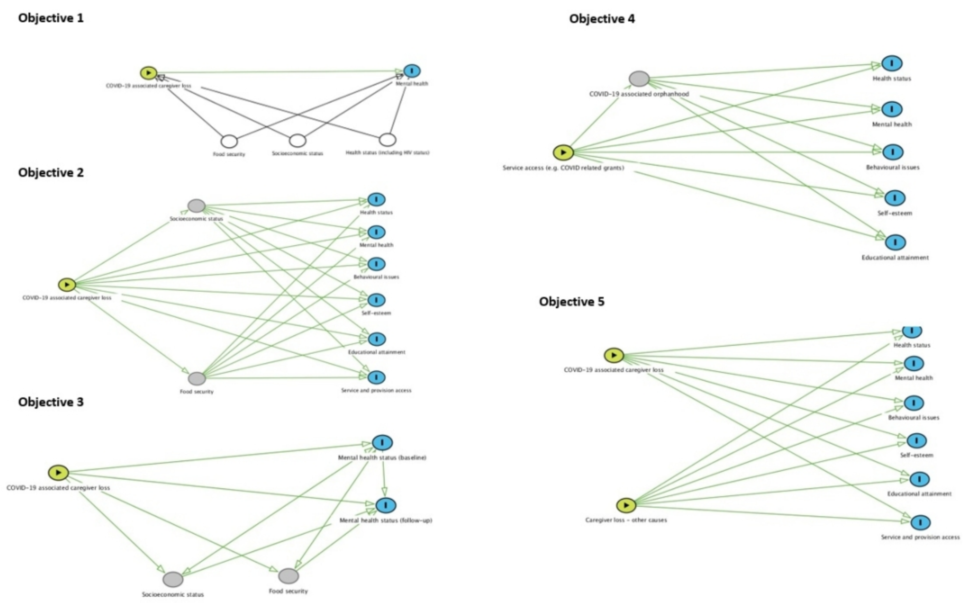

Figure 1. Directed Acyclic Graphs illustrative of the OCAY study objectives (for illustrative purposes only)

106x64mm (300 x 300 DPI)

STROBE Statement—Checklist of items that should be included in reports of ***cohort studies***

	Item No	Recommendation	Page No
**Title and abstract**	1	(*a*) Indicate the study's design with a commonly used term in the title or the abstract<br>(*b*) Provide in the abstract an informative and balanced summary of what was done and what was found	1,2
**Introduction**			
Background/rationale	2	Explain the scientific background and rationale for the investigation being reported	3
Objectives	3	State specific objectives, including any prespecified hypotheses	3
**Methods**			
Study design	4	Present key elements of study design early in the paper	4
Setting	5	Describe the setting, locations, and relevant dates, including periods of recruitment, exposure, follow-up, and data collection	3
Participants	6	(*a*) Give the eligibility criteria, and the sources and methods of selection of participants. Describe methods of follow-up<br>(*b*) For matched studies, give matching criteria and number of exposed and unexposed	4
Variables	7	Clearly define all outcomes, exposures, predictors, potential confounders, and effect modifiers. Give diagnostic criteria, if applicable	4
Data sources/ measurement	8*	For each variable of interest, give sources of data and details of methods of assessment (measurement). Describe comparability of assessment methods if there is more than one group	5-8
Bias	9	Describe any efforts to address potential sources of bias	9
Study size	10	Explain how the study size was arrived at	9
Quantitative variables	11	Explain how quantitative variables were handled in the analyses. If applicable, describe which groupings were chosen and why	9
Statistical methods	12	(*a*) Describe all statistical methods, including those used to control for confounding<br>(*b*) Describe any methods used to examine subgroups and interactions<br>(*c*) Explain how missing data were addressed<br>(*d*) If applicable, explain how loss to follow-up was addressed<br>(*e*) Describe any sensitivity analyses	9
**Results**			n/a
Participants	13*	(a) Report numbers of individuals at each stage of study—eg numbers potentially eligible, examined for eligibility, confirmed eligible, included in the study, completing follow-up, and analysed<br>(b) Give reasons for non-participation at each stage<br>(c) Consider use of a flow diagram	
Descriptive data	14*	(a) Give characteristics of study participants (eg demographic, clinical, social) and information on exposures and potential confounders<br>(b) Indicate number of participants with missing data for each variable of interest<br>(c) Summarise follow-up time (eg, average and total amount)	
Outcome data	15*	Report numbers of outcome events or summary measures over time	

For peer review only

For peer review only - http://bmjopen.bmj.com/site/about/guidelines.xhtml

Main results	16	(*a*) Give unadjusted estimates and, if applicable, confounder-adjusted estimates and their precision (eg, 95% confidence interval). Make clear which confounders were adjusted for and why they were included<br>(*b*) Report category boundaries when continuous variables were categorized<br>(*c*) If relevant, consider translating estimates of relative risk into absolute risk for a meaningful time period	
Other analyses	17	Report other analyses done—eg analyses of subgroups and interactions, and sensitivity analyses	
**Discussion**			
Key results	18	Summarise key results with reference to study objectives	
Limitations	19	Discuss limitations of the study, taking into account sources of potential bias or imprecision. Discuss both direction and magnitude of any potential bias	
Interpretation	20	Give a cautious overall interpretation of results considering objectives, limitations, multiplicity of analyses, results from similar studies, and other relevant evidence	
Generalisability	21	Discuss the generalisability (external validity) of the study results	
**Other information**			
Funding	22	Give the source of funding and the role of the funders for the present study and, if applicable, for the original study on which the present article is based	

*Give information separately for exposed and unexposed groups.

Note: An Explanation and Elaboration article discusses each checklist item and gives methodological background and published examples of transparent reporting. The STROBE checklist is best used in conjunction with this article (freely available on the Web sites of PLoS Medicine at http://www.plosmedicine.org/, Annals of Internal Medicine at http://www.annals.org/, and Epidemiology at http://www.epidem.com/). Information on the STROBE Initiative is available at http://www.strobe-statement.org.

For peer review only
